# Use of Data-Driven Methods to Predict Long-term Patterns of Health Care Spending for Medicare Patients

**DOI:** 10.1001/jamanetworkopen.2020.20291

**Published:** 2020-10-19

**Authors:** Julie C. Lauffenburger, Mufaddal Mahesri, Niteesh K. Choudhry

**Affiliations:** 1 Center for Healthcare Delivery Sciences, Department of Medicine, Brigham and Women’s Hospital, Boston, Massachusetts; 2 Division of Pharmacoepidemiology and Pharmacoeconomics, Department of Medicine, Brigham and Women’s Hospital, Harvard Medical School, Boston, Massachusetts

## Abstract

**Importance:**

Current approaches to predicting health care costs generally rely on a single composite value of spending and focus on short time horizons. By contrast, examining patients’ spending patterns using dynamic measures applied over longer periods may better identify patients with different spending and help target interventions to those with the greatest need.

**Objective:**

To classify patients by their long-term, dynamic health care spending patterns using a data-driven approach and assess the ability to predict spending patterns, particularly using characteristics that are potentially modifiable through intervention.

**Design, Setting, and Participants:**

This cohort study used a retrospective cohort design from a random nationwide sample of Medicare fee-for-service administrative claims data to identify beneficiaries aged 65 years or older with continuous eligibility from 2011 to 2013. Statistical analysis was performed from August 2018 to December 2019.

**Main Outcomes and Measures:**

Group-based trajectory modeling was applied to the claims data to classify the Medicare beneficiaries by their total health care spending patterns over a 2-year period. The ability to predict membership in each trajectory spending group was assessed using generalized boosted regression, a data mining approach to model building and prediction, with split-sample validation. Models were estimated using (1) prior-year predictors and (2) prior-year predictors potentially modifiable through intervention measured in the claims data. These models were evaluated using validated C-statistics. The relative influence of individual predictors in the models was evaluated.

**Results:**

Among the 329 476 beneficiaries, the mean (SD) age was 76.0 (7.2) years and 190 346 (57.8%) were female. This final 5-group model included a minimal-user group (group 1, 37 572 individuals [11.4%]), a low-cost group (group 2, 48 575 individuals [14.7%]), a rising-cost group (group 3, 24 736 individuals [7.5%]), a moderate-cost group (group 4, 83 338 individuals [25.3%]), and a high-cost group (group 5, 135 255 individuals [41.2%]). Potentially modifiable characteristics strongly predicted these patterns (C-statistics range: 0.68-0.94). For groups with progressively increasing spending in particular, the most influential factors were number of medications (relative influence: 29.2), number of office visits (relative influence: 30.3), and mean medication adherence (relative influence: 33.6).

**Conclusions and Relevance:**

Using a data-driven approach, distinct spending patterns were identified with high accuracy. The potentially modifiable predictors of membership in the rising-cost group represent important levers for early interventions that may prevent later spending increases. This approach could be adapted by organizations to target quality improvement interventions, particularly because numerous health care organizations are increasingly using these routinely collected data.

## Key Points

**Question:**

What are the long-term spending patterns by Medicare beneficiaries, and do baseline patient factors that are potentially modifiable predict these patterns?

**Findings:**

In this cohort study using a data-driven approach to classifying Medicare beneficiaries by their spending over 2 years, 5 patterns were identified and could be predicted, including those with consistent spending levels and others with spending that increased progressively. The most influential potentially modifiable factors were number of medications, number of office visits, and mean medication adherence.

**Meaning:**

These findings suggest that spending by Medicare beneficiaries falls into 5 distinct groups and could be accurately predicted; this approach could be adapted by organizations to target interventions.

## Teaser

This cohort study uses group-based trajectory modeling to assess the spending patterns of Medicare beneficiaries over a 2-year period and explores whether such a model can predict spending patterns using potentially modifiable patient characteristics.

## Introduction

With health care spending now accounting for almost 18% of the US gross domestic product, identifying individuals who may benefit from interventions to address potentially avoidable spending has become a central priority for health insurers and health care professionals.^1^ Current approaches generally focus on prediction or intervention for patients who may have escalating costs on the basis of a single composite value of total spending over short time periods.^2,3^

However, many patients experience substantial increases or decreases in spending not captured by these approaches.^4,5,6,7,8,9^ For example, Tamang et al^10^ identified a definable group of low-spending patients in 1 year whose costs bloomed (ie, they became high-spending individuals) in the subsequent year in Denmark. Similarly, Lauffenburger et al^11^ observed 7 distinct, dynamic patterns of spending over a 1-year period in commercially insured beneficiaries, including individuals whose costs increased rapidly toward the end of the year and another group of high-cost individuals for whom spending decreased.

These prior studies were conducted over a 1-year period, yet there may also be dynamic patterns of spending over longer periods that may have implications both for whom to outreach for intervention and when to do so.^1,12^ For example, patients with the same clinical conditions who are hospitalized early during a 12-month period may differ meaningfully from those hospitalized later, although both could be identified as having rising costs.^13,14^ If these different spending patterns could be predicted using routinely collected data, then the ability to better proactively differentiate patients with increasing or decreasing spending patterns could better target interventions to those who are at greatest need of improved health or cost containment.^15^ The predictive accuracy of spending may also be higher when evaluating a long-term, compared with a short-term, time horizon as seen for other outcomes.^16^ Accordingly, we sought to classify patients according to their spending patterns over a 2-year period and to evaluate the ability to predict these spending groups using patient characteristics that are potentially modifiable.

## Methods

This cohort study was approved by the institutional review board of Brigham and Women’s Hospital and was granted a waiver of informed patient consent because the data are secondary routinely collected data. This study follows reporting requirements of the Strengthening the Reporting of Observational Studies in Epidemiology (STROBE) reporting guideline.

### Setting and Study Design

This study used administrative claims data from a 1-million-member sample of Medicare fee-for-service beneficiaries; the original sample included approximately 20 000 beneficiaries in a nationwide quality improvement program and approximately 980 000 randomly selected patients nationally.^17^ We restricted the cohort to the randomly selected patients and used their paid Medicare Parts A, B, and D patient-level files containing all procedures, physician encounters, hospitalizations, and filled outpatient prescriptions, including amounts paid by the insurer and patient. These data were linked to eligibility data including age, race/ethnicity, gender, and geographic location of residence. Aggregate zip code level data on median income and educational attainment were obtained by linking with 2010 US Census data.

To be included, patients had to be aged 65 years or older and maintain continuous eligibility from January 1, 2011, to December 31, 2013. The cohort entry date was defined as January 1, 2012, to provide 1 year of prior year of baseline data (year 0) and 2 years of follow-up data (year 1 and year 2) (eFigure 1 in the Supplement).

### Costs

We measured total monthly health care spending over a 2-year period for each patient by summing the allowed amounts on all inpatient, outpatient, and prescription drug claims. Monthly costs were generated by summing the costs in each month and were standardized by dividing the summed costs by the number of days in that month and then multiplying the result by 30. Costs were then logarithmically transformed to normalize their distribution, after adding $0.01, as frequently done.^9,18^ Costs were inflated using the Medical Care Component of the Consumer Price Index to 2013 dollars when necessary.

### Predictors

Using data from Medicare enrollment files and claims, we defined 37 clinically relevant baseline characteristics that were potential predictors of future spending (eTable 1 in the Supplement). These baseline variables were measured during the 12 months prior to the 2-year period during which cost outcomes were evaluated (eFigure 1 in the Supplement). These variables were based on characteristics used in cost modeling in claims data in the peer-reviewed literature and from the quality-cost theoretical framework.^6,10,11,15^ These sets of predictors have also been shown to have equivalent predictive accuracy of predicting 1-year spending as proprietary risk-adjustment methods.^11^

Sociodemographic characteristics included age, race/ethnicity, gender, and community-level variables based on member’s zip code of residence, including median household income and educational attainment. Clinical comorbidities were measured using *International Classification of Diseases, Ninth Revision* codes (eAppendix and eTable 1 in the Supplement). Each patients’ number of unique prescriptions by generic name (ie, therapeutic complexity), physician office visits, emergency department visits, hospitalizations, unique physicians visited, unique pharmacies used, benefits’ generosity^19^ (copayments and deductibles or total net payments), and baseline year total costs were also measured. Adherence to long-term medication classes (eg, β-blockers) was measured in the baseline year.^11^ For each class, we created a supply diary beginning with the first fill for each class in the baseline year. This diary linked all observed fills based on dispensing date and days’ supply; switching was allowed within each class (eg, β-blockers). From this, we calculated the proportion of days covered (PDC) as a mean across classes that the patient filled to yield 1 mean PDC.^20,21^

We categorized each predictor by whether it was potentially modifiable, defined by whether it could theoretically be addressed in interventions and by classifications in prior literature.^22,23^ For example, number of unique physicians could be potentially modifiable, while race/ethnicity is not. In total, we classified 10 predictors as potentially modifiable (Table 1).

**Table 1.** Patient Characteristics by Spending Trajectory

Covariates	Patients, No. (%)				
	Group 1: minimal user (n = 37 572)	Group 2: low cost (n = 48 575)	Group 3: rising cost (n = 24 736)	Group 4: moderate cost (n = 83 338)	Group 5: high cost (n = 135 255)
Demographic characteristics					
Age, mean (SD), y	73.8 (7.7)	74.8 (6.8)	75.1 (6.9)	75.9 (7.0)	77.1 (7.2)
Female	16 394 (43.6)	26 531 (54.6)	13 500 (54.6)	49 287 (59.1)	84 634 (62.6)
Race/ethnicity					
Non-Hispanic White	30 732 (81.8)	42 723 (88.0)	22 020 (89.0)	74 184 (89.0)	118 637 (87.7)
Black	3610 (9.6)	3255 (6.7)	1169 (6.6)	5332 (6.4)	9646 (7.1)
Other	1299 (3.5)	1297 (2.7)	539 (2.2)	1588 (1.9)	2293 (1.7)
Asian or Pacific Islander	867 (2.3)	792 (1.6)	322 (1.3)	1330 (1.6)	2448 (1.8)
Hispanic	1064 (2.8)	508 (1.1)	236 (1.0)	904 (1.1)	2231 (1.7)
Zip code median income, mean (SD), $	59 960 (24 347)	56 572 (24 199)	56 696 (23 765)	56 683 (23 776)	55 929 (23 808)
Zip code high school graduates, mean (SD), %	80.8 (21.0)	84.4 (16.6)	84.5 (16.2)	84.6 (15.8)	83.9 (15.9)
Health care use					
Part D					
Plan switch	163 (0.4)	173 (0.4)	82 (0.3)	468 (0.6)	1828 (1.4)
Low-income subsidy	3584 (9.5)	2735 (5.6)	1379 (5.6)	8063 (9.7)	31 019 (22.9)
Office visits, mean (SD), No.^a^	1.2 (2.0)	4.5 (3.5)	4.7 (3.7)	7.1 (5.0)	11.3 (8.3)
Physicians, mean (SD), No.^a^	0.4 (0.7)	1.0 (1.0)	1.0 (0.9)	1.3 (1.1)	1.8 (1.3)
Pharmacies used, mean (SD), No.^a^	0.1 (0.4)	0.4 (0.8)	0.3 (0.7)	0.8 (1.1)	1.3 (1.3)
Hospitalizations, mean (SD), No.	0.0 (0.2)	0.1 (0.4)	0.1 (0.3)	0.2 (0.5)	0.4 (0.8)
Emergency department visits, mean (SD), No.^a^	0.1 (0.4)	0.2 (0.6)	0.2 (0.6)	0.3 (0.7)	0.6 (1.3)
Unique drugs, mean (SD), No.^a^	0.2 (1.1)	1.0 (2.2)	0.9 (2.2)	3.1 (3.9)	8.0 (7.0)
Prescription generosity, mean (SD)	0.1 (0.2)	0.1 (0.3)	0.1 (0.2)	0.2 (0.3)	0.2 (0.2)
Medical benefits’ generosity, mean (SD)	0.2 (0.3)	0.2 (0.2)	0.2 (0.2)	0.1 (0.1)	0.1 (0.8)
Total baseline year costs, mean (SD), $	1629 (5948)	4969 (10 296)	4762 (8989)	8314 (13 052)	19 941 (26 331)
Long-term medication use	1261 (3.4)	7942 (16.4)	3445 (13.9)	35 142 (42.2)	88 922 (65.7)
Medication adherence, mean (SD)^a^	0.55 (0.30)	0.78 (0.24)	0.76 (0.25)	0.82 (0.19)	0.82 (0.18)
Comorbidities					
Comorbidity score, mean (SD)	0.1 (0.9)	0.3 (1.4)	0.3 (1.4)	0.7 (1.8)	2.1 (2.7)
Coronary artery disease	312 (0.8)	1065 (2.2)	518 (2.1)	3209 (3.9)	13 664 (10.1)
Prior myocardial infarction	55 (0.2)	171 (0.4)	66 (0.3)	430 (0.5)	1491 (1.1)
Asthma or chronic obstructive pulmonary disease	1659 (4.4)	5047 (10.4)	2952 (11.9)	12 795 (15.4)	40 073 (29.6)
Hypertension	8962 (23.9)	30 172 (62.1)	15 683 (63.4)	63 097 (75.7)	115 869 (85.7)
Diabetes	577 (1.5)	2508 (5.2)	1360 (5.5)	7857 (9.4)	25 653 (19.0)
Acute kidney failure or end stage kidney disease	197 (0.5)	555 (1.1)	275 (1.1)	1591 (1.9)	8604 (6.4)
Dementia	210 (0.6)	555 (1.4)	362 (1.5)	1162 (1.9)	7805 (5.8)
Depression^a^	519 (1.4)	2120 (4.4)	1188 (4.8)	5878 (7.1)	20 787 (15.4)
Stroke	93 (0.3)	224 (0.5)	102 (0.4)	628 (0.8)	2165 (1.6)
Liver disease	28 (0.1)	62 (0.1)	15 (0.1)	184 (0.2)	702 (0.5)
Congestive heart failure	107 (0.3)	325 (0.7)	168 (0.7)	1083 (1.3)	7235 (5.4)
Hyperlipidemia	7821 (20.8)	30 098 (62.0)	15 376 (62.2)	60 720 (72.9)	105 003 (77.6)
Atrial fibrillation	129 (0.3)	420 (0.9)	216 (0.9)	1607 (1.9)	8130 (6.0)
Osteoporosis	1839 (4.9)	8562 (17.6)	4304 (17.4)	19 080 (22.9)	38 204 (28.3)
Obesity^a^	511 (1.3)	1867 (3.8)	971 (3.9)	4572 (5.5)	13 223 (9.8)
Acute stress^a^	245 (0.7)	780 (1.6)	385 (1.6)	1973 (2.4)	7427 (5.5)
Tobacco use^a^	1156 (3.1)	2851 (5.9)	1474 (6.0)	6094 (7.3)	16 499 (12.2)

^a^ Denotes potentially modifiable predictors.

### Data-Driven Approach to Modeling Long-term Costs

We used trajectory modeling to empirically classify spending during follow-up. One advantage is that it allows the data to define the cost outcomes, rather than using arbitrarily selected thresholds.^24^ It also considers changes in spending over time, rather than aggregating costs over a set time.^25^ To define spending patterns, we used the previously described SAS procedure Proc Traj, a free add-on.^24,25,26^ In brief, group-based trajectory models are an application of finite mixture modeling that identify clusters of individuals with similar outcome patterns over time.^24^ This modeling approach analyzes longitudinal data by fitting a semiparametric (discrete) mixture model, estimating each individual’s probability of membership in each group, and assigning them to the group according to their highest probability. We modeled longitudinal cost trajectories using calendar month as the time variable, costs in each month, order equal to 4, and a censored-normal distribution (linear between minimum and maximum values).^11,24,26^

The models were estimated using a forward classifying approach using 2 to 7 groups, each time investigating model fit using the bayesian information criterion (BIC), whereby a lower BIC indicates better model fit.^24^ The number of groups investigated was capped at 7 on the basis of groupings observed in prior work.^11^ In addition to considering BIC, other key considerations in selecting the best-fitting trajectory were the ability to visually interpret separate groups, minimum membership probabilities in each group, and having 5% or more of the sample in each group.^26,27,28^

### Statistical Analysis

After selecting the best fitting number of trajectories, we assessed the ability to predict membership in each 2-year trajectory group using boosted logistic regression, a nonparametric machine learning method. The boosted algorithm is considered one of the best data-mining approaches for prediction problems.^16,29^ Specifically, the algorithm creates a prediction model by building numerous small regression trees that together provide highly accurate classification.^30^ The boosting algorithm has several built-in protections from model overfitting, provides automatic variable selection, and describes the relative influence of predictors.^31^ They also consider all possible interaction terms between potential predictors. We used the gbm package in R with 5-fold cross-validation to identify the optimal number of trees and applied standard default values for tuning parameters to identify the optimal model.^16^

For each trajectory group, we estimated 2 separate models. The first included all 37 baseline predictors (model 1) and the second included only the 10 baseline predictors that were considered a priori to be potentially modifiable (model 2). Because of the ability of boosted regression to handle missing data, an indicator of long-term medication use and mean PDC were both included as variables for model 1, and mean PDC was included alone as a variable for model 2.

To avoid overoptimism bias, we used internal split-sample validation by randomly dividing the full cohort into 2 halves as an initial derivation sample and a validation sample for all models.^32^ We evaluated each model through discrimination measures.^33^ Discrimination, the model’s ability to distinguish between patients who do and do not experience the outcome, was measured by the C-statistic, which ranges from 0.5 (noninformative model) to 1.0 (perfect prediction).^34,35^

For clinical context, we explored the association between potentially modifiable baseline characteristics and membership in a rising-cost trajectory compared with other trajectory groups that had similar spending at baseline. Specifically, we used multivariable logistic regression to compare membership in the rising-cost trajectory, including each potentially modifiable variable vs other groups. This approach provides insight into baseline factors that may help distinguish patients who become costly later (ie, at least a year later) and potential levers for interventions. We also explored the relative influence of each potentially modifiable predictor from model 2.

We also evaluated the ability to predict patients who experience rising costs in year 2 defined using a decile-threshold approach (ie, those in the lower 90% of spending in year 1 and then were in the top 10% of spending in year 2^10^) and patients who in trajectory modeling were estimated as belonging to a rising-cost trajectory. For this approach, we estimated each outcome with 2 additional models with boosted regression. Model 3 used all baseline predictors, and model 4 used the potentially modifiable predictors. This approach helps provide insight into whether these spending increases could be accurately predicted using baseline information less temporal to the spending changes, which could ultimately inform intervention design and allow more time for them to be implemented.

We conducted several sensitivity analyses. Although our primary analysis included zip code sociodemographic characteristics, we also included patients’ region of residence based on enrollment files as a predictor in model 1. Then, we included adherence to each class separately as predictors in models 1 and 2. Finally, we repeated measurements and analyses in a subsequent year (ie, 2012-2014) to confirm generalizability (eAppendix in the Supplement).

All analyses except for the boosted regression were performed using SAS version 9.4 (SAS Institute); the boosting algorithm was performed using R version 3.4.1 (The R Project for Statistical Computing). Statistical analysis was performed from August 2018 to December 2019.

## Results

### Study Population and Characteristics

Our cohort consisted of 329 476 patients (eTable 2 in the Supplement). Their mean (SD) age was 76.0 (7.2) years, and 190 346 (57.8%) were women. A 5-group trajectory model best described the 2-year spending patterns (Figure); the model on the log scale is shown in eFigure 2 in the Supplement. The probabilities of group membership are in eTable 3 in the Supplement. Trajectories with alternative numbers of groups and corresponding BICs are shown in eFigure 3 in the Supplement; models with more groups had marginal improvements and were less interpretable.

**Figure.**
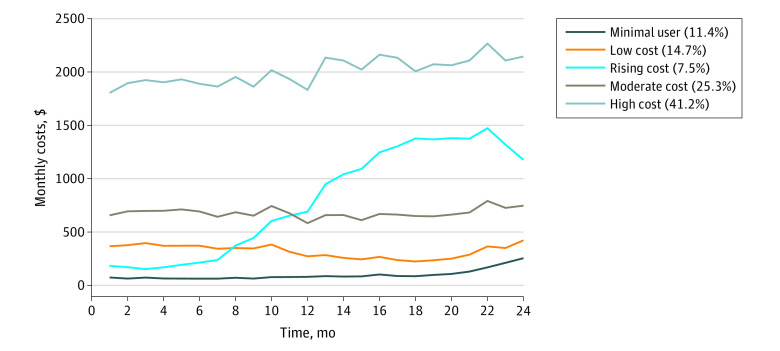
2-Year Spending Patterns Using Trajectory Modeling The mean observed spending levels using 5-group trajectory modeling in the full sample are plotted. The percentages in the key refer to the number of patients who belong to each trajectory group out of the full cohort (bayesian information criterion for this model: 21704747).

This final 5-group model included a minimal-user group (group 1, 37 572 individuals [11.4%]), a low-cost group (group 2, 48 575 individuals [14.7%]), a rising-cost group (group 3, 24 736 individuals [7.5%]), a moderate-cost group (group 4, 83 338 individuals [25.3%]), and a high-cost group (group 5, 135 255 individuals [41.2%]). Baseline characteristics for each group are shown in Table 1.

### Cost Prediction

Table 2 shows the results of the main prediction models in the validation sample. Four of the 5 2-year spending trajectory groups could be accurately predicted using all baseline predictors, especially the minimal-user (C-statistic: 0.951), low-cost (C-statistic: 0.810), rising-cost (C-statistic: 0.764), and high-cost groups (C-statistic: 0.899). Using potentially modifiable predictors alone, overall predictive ability remained moderate to strong, with the exception of the moderate-cost group (eg, C-statistic: 0.684).

**Table 2.** Ability of Models to Predict 2-Year Spending Trajectory Groups

Group	Validation C-statistic
All baseline predictors, model 1	
Group 1: minimal user	0.951
Group 2: low cost	0.810
Group 3: rising cost	0.764
Group 4: moderate cost	0.728
Group 5: high cost	0.899
Potentially modifiable predictors, model 2	
Group 1: minimal user	0.942
Group 2: low cost	0.783
Group 3: rising cost	0.753
Group 4: moderate cost	0.684
Group 5: high cost	0.873

Table 3 shows potentially modifiable prior-year predictors of being in a rising-cost trajectory compared with the other 3 groups with similar spending in the prior baseline year (mean, $1500-$8000 in year 0). In particular, using more medications (odds ratio [OR]: 0.81; 95% CI, 0.79-0.84) and having more office visits (OR: 0.98; 95% CI, 0.97-0.99) were associated with lower odds of being in the rising-cost trajectory. Seeing more physicians (OR: 1.04; 95% CI, 1.02-1.06) and using tobacco (OR: 1.10; 95% CI, 1.02-1.20) were also factors independently associated with rising-cost membership. eFigure 4 in the Supplement shows the relative influence plots for each group incorporating only potentially modifiable characteristics (model 2). The plot for predicting the rising-cost group in particular indicates that the most predictive potentially modifiable factors were mean medication adherence (relative influence: 33.6), number of office visits (relative influence: 30.3), and number of medications (relative influence: 29.2).

**Table 3.** Association Between Potentially Modifiable Factors and Membership in the Rising-Cost Spending Trajectory (Group 3) vs Other Trajectory Groups^a^

Characteristics	OR (95% CI) for group 3: rising cost
Intercept (SE)	−1.86 (0.02)
Baseline covariate	
Unique medications, No.^b^	0.81 (0.79-0.84)
Office visits, No.^b^	0.98 (0.97-0.99)
Physicians, No.^b^	1.04 (1.02-1.06)
Pharmacies, No.^b^	0.99 (0.95-1.02)
Emergency department visits, No.^b^	0.98 (0.94-1.01)
Depression	1.01 (0.92-1.10)
Tobacco use	1.10 (1.02-1.20)
Obesity	1.08 (0.98-1.19)
Acute stress	0.87 (0.74-1.02)

Abbreviation: OR, odds ratio.

^a^ Conducted within validation sample using logistic regression model with only potentially modifiable covariates compared with groups 1, 2, and 4.

^b^ Odds ratios are presented as a 1-unit increase for continuous variables.

The results from the models predicting rising costs using a decile-threshold–based method and the trajectory group method are shown in eTable 4 in the Supplement. Patients in the decile-threshold–based approach had higher total 2-year costs on average ($39 737), compared with the trajectory approach ($23 670). The ability to predict decile-threshold–based rising costs (model 4 C-statistic: 0.643) was lower than the trajectory-based approach (model 4 C-statistic: 0.753).

Sensitivity analyses incorporating region of residence and medication adherence to by class are shown in eTables 5 and 6 in the Supplement. Notably, trajectory group membership was fairly similar across regions, and including these predictors did not meaningfully change C-statistics. Replication in a subsequent year of data resulted in similar patterns and sizes of group membership (eFigure 5 in the Supplement) as well as ability to predict those groups (eTable 7 in the Supplement).

## Discussion

Using a data-driven approach to classify 2-year health spending for Medicare beneficiaries, we observed 5 distinct spending patterns. Membership in these groups could be accurately predicted, even when using a simple set of potentially modifiable characteristics from claims data. These results suggest that this approach could potentially help inform the design, application, and timing of interventions.

Prior efforts to predict health care spending have generally focused on a single composite value, such as total yearly costs or a threshold-based measure, such as being in the top 5% of spending, both of which collapse an entire year’s spending into a static variable. These approaches have had modest accuracy; C-statistics for threshold-based outcomes have generally ranged from 0.6 to 0.8.^2,5,36,37^ Two recently published approaches offer other cluster-based solutions to elucidate subgroups of high-cost patients with some notable successes.^38,39^ However, these were not applied to evaluate changes in spending, outcomes over more than 1 year, or to elucidate patients with rising costs.^38,39^ They also focused on Medicare Advantage populations, which can differ from fee-for-service beneficiaries.^40,41^

Patients may have dynamic patterns of spending over longer periods of time that can be potentially meaningful, with implications on whom to outreach for intervention as well as when and perhaps how to do so.^1,12^ For example, Tamang et al^10^ identified low-spending patients in 1 year whose costs bloomed in the subsequent year using thresholds. When applied to our data, the ability to predict these patients using baseline data alone was modest. Using a data-driven approach, we observed a similarly sized group whose costs later increased that could be predicted somewhat better. One possible explanation could be that the 2-year time horizon itself as an outcome helped discriminate between groups. The ability to proactively differentiate between patients with rising or falling spending patterns using distally measured variables could better target interventions to those who are at greatest need. If successful, using these longer time horizons could allow more time for the implementation of potential interventions.^42^

Focusing interventions on patients with rising costs has some theoretical advantages, even though predictive ability was modest. First, the size of the group identified in this study was modest (ie, 7.5%). Of course, it still may be infeasible to intervene upon a group this large, and not all costs may be preventable. Identifying additional segmentation may be necessary, and the use of this approach may be just a starting point. Regardless, the ability to predict better could target interventions to those at greater need, and targeting has been shown to result in better population-level outcomes.^43^

When considering potential interventions, a prediction rule comprising the most influential potentially modifiable variables could be applied to better target patients. We observed several clinically actionable characteristics, such as therapeutic complexity (ie, number of medications or office visits), depression, medication adherence, and tobacco use that could be levers for interventions. Filling fewer medications and having fewer office visits were also predictors of the rising-cost trajectory, suggesting that patients may not be getting sufficient care to prevent future escalation of health problems.^22^ This information could also be used for intervention design to improve care.

Many health care organizations, insurers, researchers, and policy makers use claims data to identify patients for interventions. Therefore, the ability to better leverage these routinely collected data for cost predictions and interventions with a variety of more nuanced cost-modeling methods holds wide potential. Moreover, using data-driven approaches to classify longer-term spending may hold promise compared with threshold-based approaches alone.

### Limitations

Several limitations warrant mention. First, we examined trajectories from January to December; patients with incomplete enrollment or other policy start and end dates may differ. Because of differences in how outcomes are categorized, model performance of predicting a cost trajectory (binary outcome) cannot be directly compared with predicting total costs (continuous outcome) or patients defined by the rising-cost decile-threshold approach. The variables included in prediction models may also not be exhaustive, and although we used validated algorithms, they may be insufficiently sensitive. Trajectory modeling also provides predicted group membership; individual members may be assigned to their closest trajectory, but there could be within-group heterogeneity. The high-cost group was large, possibly because of how the model was specified (ie, log costs); one could potentially apply trajectories to identify subgroups within that group for further segmentation. Although group distribution did not differ on the basis of geographical region, the costs themselves were not adjusted for region; similarly, moving could have impacted relative changes in spending, but this was beyond the scope of this study. Furthermore, these results may not be generalizable to other payment systems, such as non–fee-for-service Medicare, Medicaid, or commercially insured beneficiaries. Although these other beneficiaries may have different spending levels, prior work has suggested similar patterns.^11^ Regardless, the same groups or predictive ability may not apply to other types of beneficiaries, and the results should be studied further to confirm reproducibility.

## Conclusions

Using trajectory modeling to examine a 2-year time horizon improved the understanding of dynamic patterns, including the identification of a group of patients with progressively increasing costs and a group of patients with consistently high spending. This approach could be potentially adapted by health care organizations to improve cost-containment efforts.

## Notes

Supplement. **eFigure 1.** Study Design **eTable 1.** Baseline Predictors of Spending Outcomes **eAppendix.** Supplemental Methods **eTable 2.** Patient Eligibility Criteria **eTable 3.** Predicted Probabilities for Each Trajectory Group **eFigure 2.** Two-Year Spending Patterns Using Trajectory Modeling: Original Log Scale **eFigure 3.** Trajectory Modeling of Two-Year Healthcare Spending Using Other Numbers of Groups **eFigure 4.** Relative Influence Plots From Boosted Regression Modeling for Predicting Trajectory Group Membership With Potentially-Modifiable Variables (Model 2) **eTable 4.** Validation C-Statistics From Models Predicting Patients With Future Rising Spending **eTable 5.** Geographic Region And Baseline Chronic Condition Medication Classes By Trajectory Group **eTable 6.** Validation C-Statistics From Sensitivity Analyses **eFigure 5.** Two-Year Spending Patterns Using Trajectory Modeling In 2013-2014 Data **eTable 7.** Ability of Models to Predict Two-Year Spending Trajectory Groups In 2013-2014 Data
