# Supplementary material for: Use of Data-Driven Methods to Predict Long-term Patterns of Health Care Spending for Medicare Patients
Source: JAMA Netw Open. 2020 Oct 19;3(10):e2020291. doi: 10.1001/jamanetworkopen.2020.20291 (PMC7573679; doi:10.1001/jamanetworkopen.2020.20291)
Supplement: Supplement. — eFigure 1. Study Design eTable 1. Baseline Predictors of Spending Outcomes eAppendix. Supplemental Methods eTable 2. Patient Eligibility Criteria eTable 3. Predicted Probabilities for Each Trajectory Group eFigure 2. Two-Year Spending Patterns Using Trajectory Modeling: Original Log Scale eFigure 3. Trajectory Modeling of Two-Year Healthcare Spending Using Other Numbers of Groups eFigure 4. Relative Influence Plots From Boosted Regression Modeling for Predicting Trajectory Group Membership With Potentially-Modifiable Variables (Model 2) eTable 4. Validation C-Statistics From Models Predicting Patients With Future Rising Spending eTable 5. Geographic Region And Baseline Chronic Condition Medication Classes By Trajectory Group eTable 6. Validation C-Statistics From Sensitivity Analyses eFigure 5. Two-Year Spending Patterns Using Trajectory Modeling In 2013-2014 Data eTable 7. Ability of Models to Predict Two-Year Spending Trajectory Groups In 2013-2014 Data [file jamanetwopen-e2020291-s001.pdf]

Supplemental Online Content

Lauffenburger JC, Mahesri M, Choudhry NK. Use of data-driven methods to predict long-term patterns of health care spending for Medicare patients. *JAMA Netw Open*. 2020;3(10):e2020291. doi:10.1001/jamanetworkopen.2020.20291

eFigure 1. Study Design
eTable 1. Baseline Predictors of Spending Outcomes
eAppendix. Supplemental Methods
eTable 2. Patient Eligibility Criteria
eTable 3. Predicted Probabilities for Each Trajectory Group
eFigure 2. Two-Year Spending Patterns Using Trajectory Modeling: Original Log Scale
eFigure 3. Trajectory Modeling of Two-Year Healthcare Spending Using Other Numbers of Groups
eFigure 4. Relative Influence Plots From Boosted Regression Modeling for Predicting Trajectory Group Membership With Potentially-Modifiable Variables (Model 2)
eTable 4. Validation C-Statistics From Models Predicting Patients With Future Rising Spending
eTable 5. Geographic Region And Baseline Chronic Condition Medication Classes By Trajectory Group
eTable 6. Validation C-Statistics From Sensitivity Analyses
eFigure 5. Two-Year Spending Patterns Using Trajectory Modeling In 2013-2014 Data
eTable 7. Ability of Models to Predict Two-Year Spending Trajectory Groups In 2013-2014 Data

This supplemental material has been provided by the authors to give readers additional information about their work.

© 2020 Lauffenburger JC et al. *JAMA Network Open.*

eFigure 1. Study Design

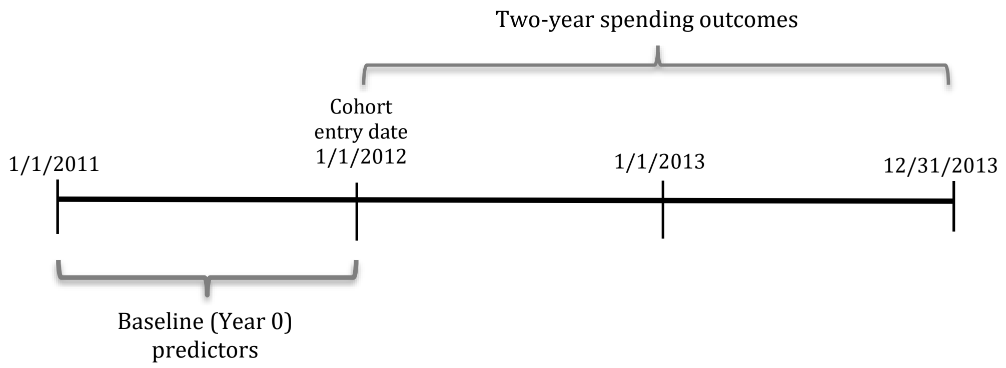

© 2020 Lauffenburger JC et al. *JAMA Network Open.*

eTable 1. Baseline Predictors of Spending Outcomes

Type and Timing	Predictors and relevant International Classification of Diseases, 9th Edition codes (ICD-9) or Current Procedural Terminology (CPT) codes
**Demographic**	
Age	Measured in enrollment files
Sex	Measured in enrollment files
Race/ethnicity	Measured in enrollment files
Median household income	Medication household income in the beneficiary's zip code (measured via 2010 US Census files), rounded to nearest US dollar
High school graduate	Percentage of persons graduating from high school in the beneficiary's zip code (measured via 2010 US Census files)
**Healthcare utilization**	
Part D plan switch	Patient switched their Part D plans in the baseline period, measured in enrollment files
Part D low income subsidy	Patient received a Part D low-income subsidy for at least one month in the baseline period
No. of office visits	Number of physician office visits, based on procedure codes
No. of physicians	Number of unique physicians associated with outpatient claims
No. of pharmacies used	Number of distinct pharmacies by Pharmacy ID that the patients filled medications at, defined using pharmacy claims
No. of hospitalizations	Number of all-cause hospitalizations
No. of Emergency Room visits	Number of all-cause emergency room visits
No. of unique drugs (i.e., therapeutic complexity)	Number of unique medications filled, by generic name
Prescription generosity	Out-of-pocket drug costs for filled medications divided by net total drug payments (Artz MB, et al. *Am J Pub Health.* 2002;92:1257-1263.)
Medical benefits' generosity	Out-of-pocket costs for all services and procedures divided by net total payments
Total baseline year costs	Total costs from all services and procedures related to inpatient and outpatient visits, procedures, durable medical equipment, home health, and medications
Chronic medication use	Defined by filling at least one medication different chronic medication classes, including medications for the following disease states: anti-hypertensive, lipid-lowering, anti-diabetic, osteoporosis, or asthma/COPD
Average adherence	Measured by proportion of days covered in the baseline period in pharmacy claims, averaged across all eligible chronic medication classes
**Comorbidities**	
Comorbidity score	Measured using the algorithm presented here: Gagne JJ et al. *J Clin Epidemiol*. 2011;64:749-759 (incorporates 37 possible comorbid conditions)
Coronary artery disease	410.x-414.x, 429.2, V45.81 (hospital discharge, any position)
Prior Myocardial infarction	410.x except 410.x2 AND length of stay >3 and <180 days (hospital discharge, any position)
Asthma or Coronary Obstructive Pulmonary Disorder	493.x, 490.x, 491.x, 492.x, 496.x
Hypertension	401.x-405.x, 437.2
Diabetes mellitus	250.x (1 inpatient or two outpatient)
Acute renal failure or End Stage Renal Disease	572.4, 580.xx, 584.xx, 580.0, 580.4, 580.89, 580.9, 582.4, 791.2, 791.3 OR 585.6
Dementia	290.0, 290.1x, 290.2x, 290.3x, 290.4x, 294.20

© 2020 Lauffenburger JC et al. *JAMA Network Open.*

Depression	311, 296.2, 296.3, 300.4, 301.12, and 309.1
Stroke	433.x1, 434 excluding 434.x0, 435.xx, 436.xx, 437.1x, 437.9x (hospital discharge, any position)
Liver disease	570-573 (esophageal varices 456.xx) (hospital discharge, any position)
Congestive heart failure	428.x, 398.91, 402.01, 402.11, 402.91, 404.01, 404.11, 404.91, 404.03, 404.13, 404.93 (hospital discharge, any position)
Hyperlipidemia	272.xx
Atrial fibrillation	427.31 (hospital discharge, any position)
Osteoporosis	733.x
Obesity	278
Acute Stress	298, 308, 309
Tobacco use	305.1, 649.0, 989.84, V15.82, CPT: 1034F

© 2020 Lauffenburger JC et al. *JAMA Network Open.*

eAppendix. Supplemental Methods

Additional detail on predictors

Clinical comorbidities were measured using International Classification of Diseases 9th edition (ICD-9) codes in medical files including comorbidities such as coronary artery disease, prior myocardial infarction, COPD/Asthma, hypertension, hyperlipidemia, congestive heart failure, stroke, major depression, diabetes, liver disease, chronic kidney disease, atrial fibrillation, Alzheimer's disease/dementia, osteoporosis, obesity, and tobacco use.

Replication in subsequent year

We used administrative claims data from the nationwide 1-million-member sample for year 2012-2014 as a separate replication dataset. As in the primary analysis, we restricted the cohort to the randomly-selected patients and used their patient-level files. To be included, patients had to be ≥65 years of age and maintain continuous eligibility from January 1, 2012 to December 31, 2014. The cohort entry date was defined as January 1, 2013 to provide 1 year of prior year baseline data ("Year 0") and 2 years of follow-up data ("Year 1" and "Year 2").

As in the primary analysis, we remeasured total monthly healthcare spending over the 2013-2014 period and used the same transformation and adjustment as described in the Methods. We also remeasured the 37 predictors using data from Medicare enrollment files and claims but in the 2012 baseline year. We classified the same 10 predictors as potentially-modifiable (asterisks denoted in Table 1).

© 2020 Lauffenburger JC et al. *JAMA Network Open.*

We also used trajectory modeling to empirically classify spending during the two-year follow-up in this replication sample. We also modeled longitudinal cost trajectories using calendar month as the time variable, costs in each month, order=4, group=5, and a censored-normal distribution (linear between minimum and maximum values). We evaluated other groupings, but the 5-group model still fit the data best.

After conducting the trajectories, as in the main paper, we also assessed the ability to predict membership in each two-year trajectory group using boosted logistic regression in this separate dataset, using the same approach described in the Methods. For each trajectory group, we estimated two separate models. The first included all 37 baseline predictors (Model 1) and the second included only the 10 baseline predictors that were considered *a priori* to be potentially-modifiable (Model 2). As the main manuscript, we used internal split-sample validation and evaluated each model through discrimination measures.

© 2020 Lauffenburger JC et al. *JAMA Network Open.*

eTable 2. Patient Eligibility Criteria

Criterion	N
Study sample	1,000,000
Was not in quality improvement study sample	976,550
Enrolled on 1/1/12 in medical and pharmacy benefits	550,215
Age ≥65 years on 1/1/11	433,561
Continuous enrollment from 1/1/11 to 12/31/13	329,476

© 2020 Lauffenburger JC et al. *JAMA Network Open.*

eTable 3. Predicted Probabilities for Each Trajectory Group

Trajectory group	Mean (SD) predicted probability of trajectory group membership	% of patients with >0.90 membership probability
Group 1: Minimal-user	0.97 (0.09)	90.4%
Group 2: Low-cost	0.91 (0.13)	73.6%
Group 3: Rising costs	0.88 (0.16)	61.6%
Group 4: Moderate cost	0.89 (0.14)	63.9%
Group 5: High cost	0.95 (0.10)	84.4%

© 2020 Lauffenburger JC et al*. JAMA Network Open.*

eFigure 2. Two-Year Spending Patterns Using Trajectory Modeling: Original Log Scale

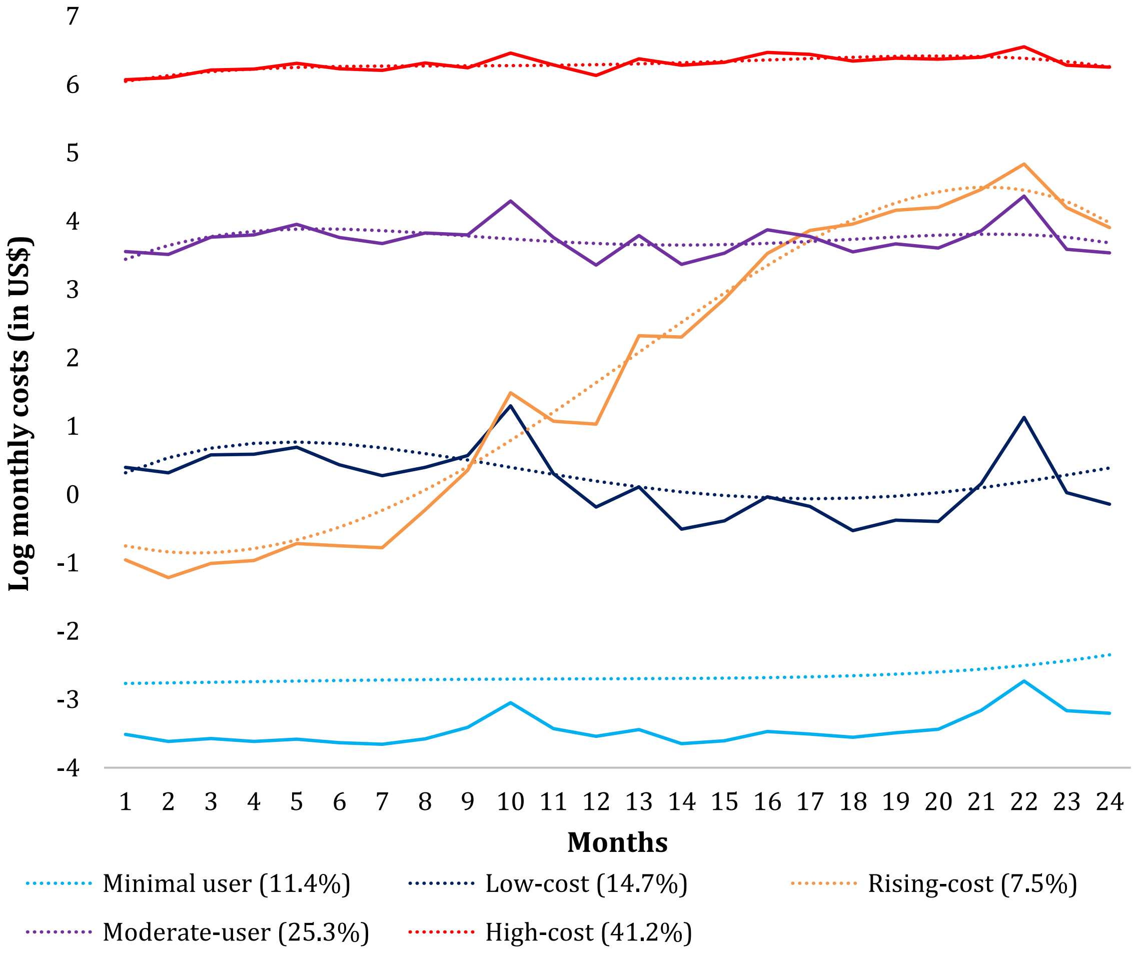

Note: The observed (solid lines) and predicted (dotted lines) probability of monthly costs on the logarithmic scale for each of 5 groups identified by the trajectory model.

© 2020 Lauffenburger JC et al. *JAMA Network Open*.

eFigure 3. Trajectory Modeling of Two-Year Healthcare Spending Using Other Numbers of Groups

A. Two-group model

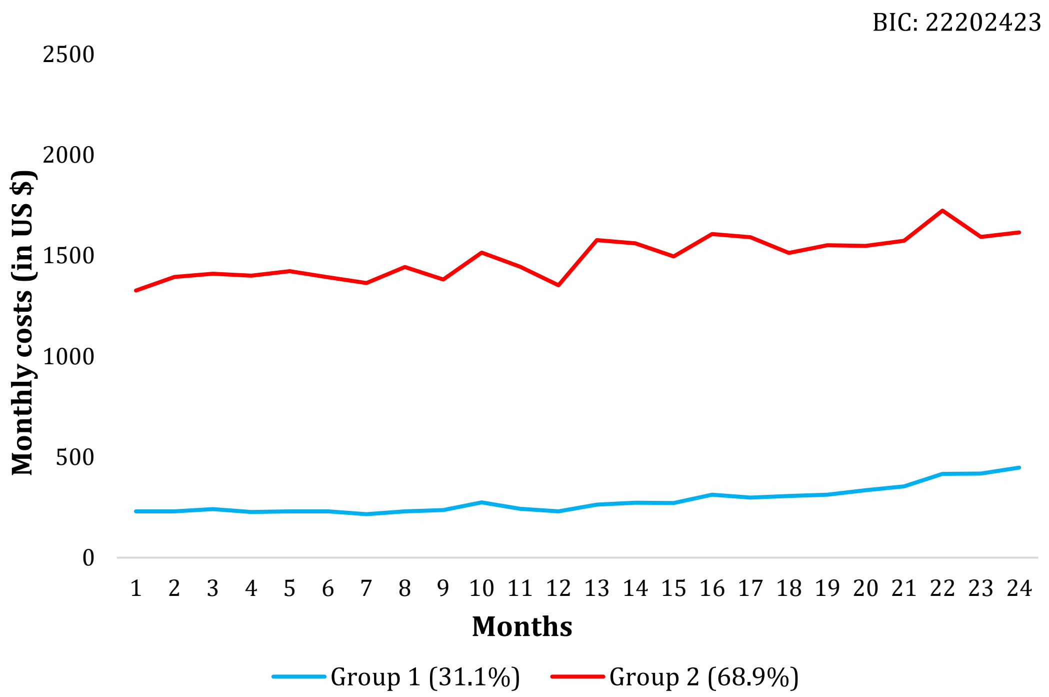

B. Three-group model

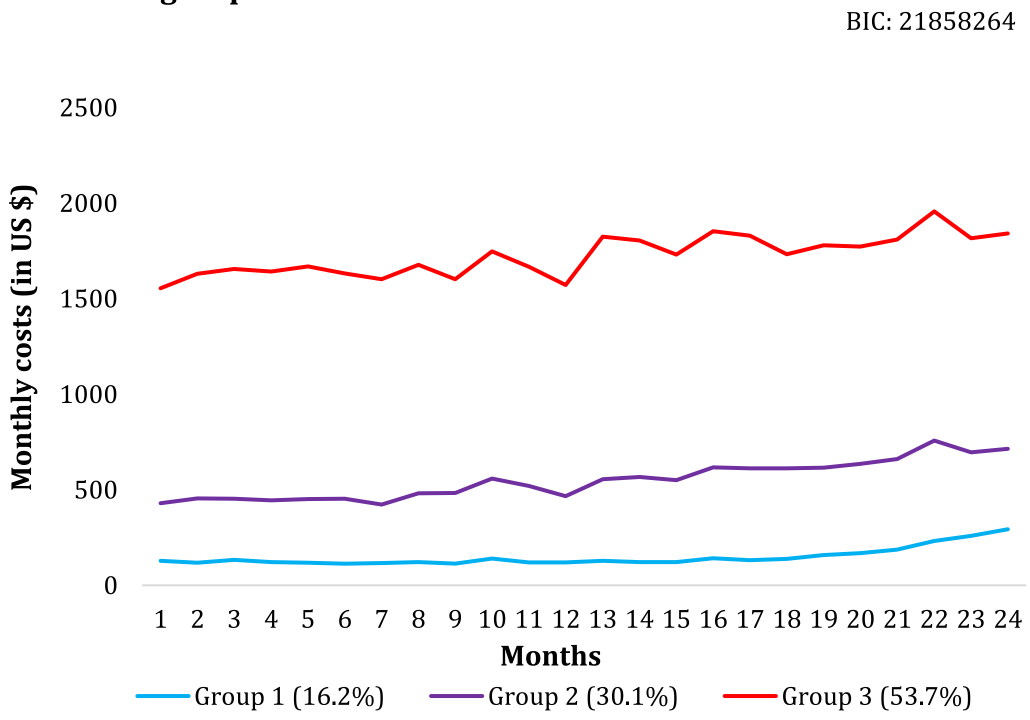

© 2020 Lauffenburger JC et al. *JAMA Network Open.*

C. Four-group model

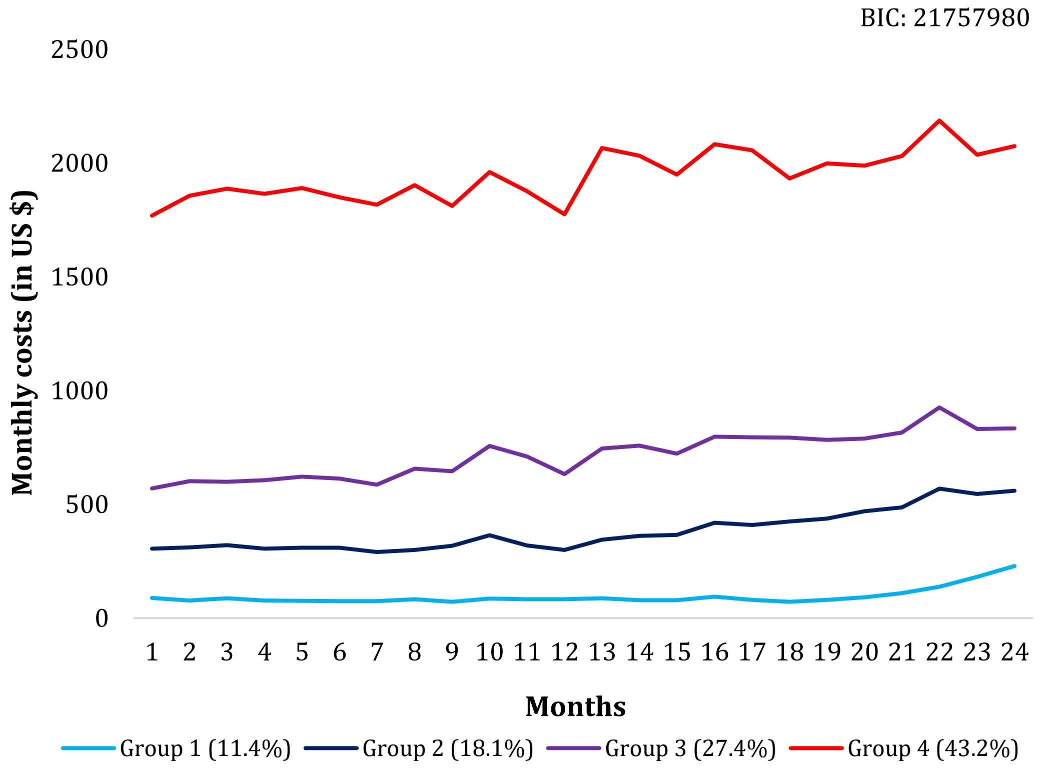

D. Six-group model

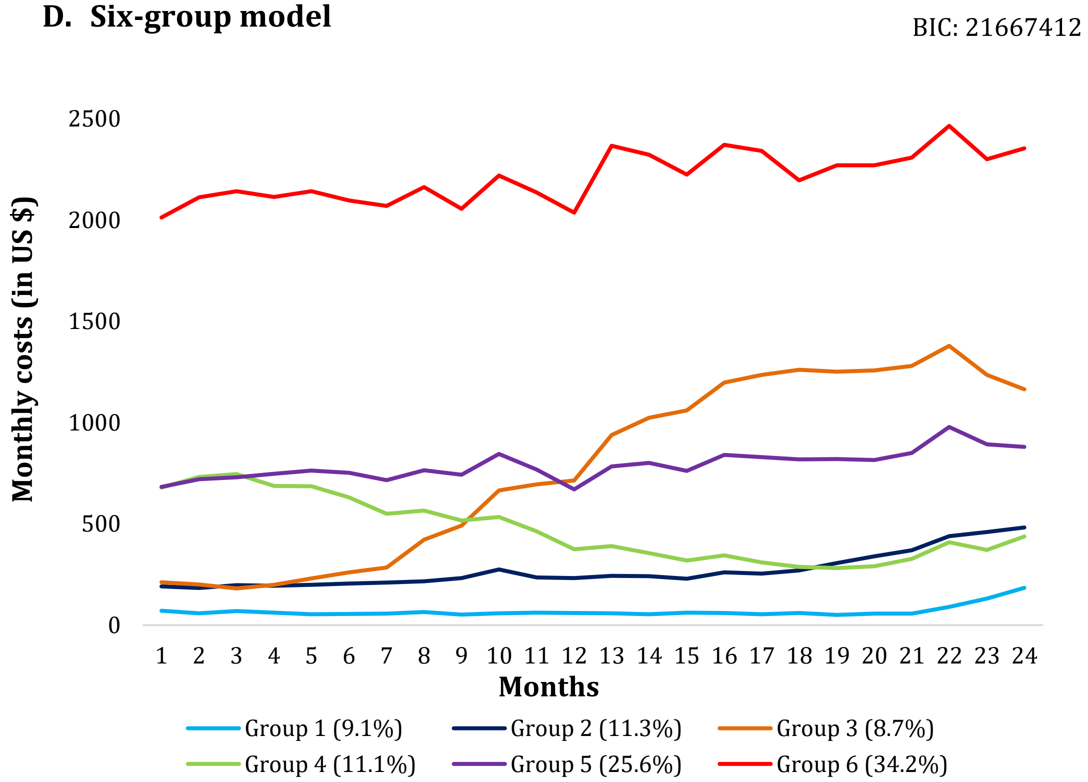

© 2020 Lauffenburger JC et al. *JAMA Network Open.*

E. Seven-group model

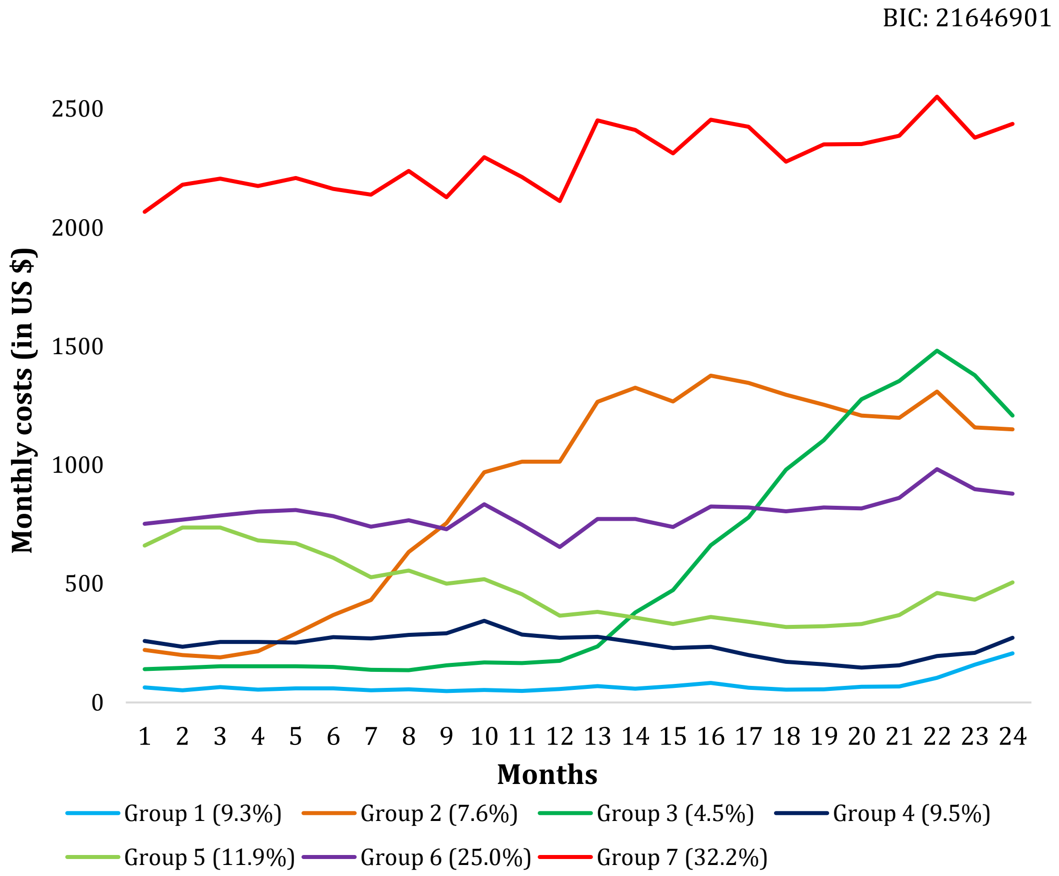

© 2020 Lauffenburger JC et al. *JAMA Network Open.*

eFigure 4. Relative Influence Plots From Boosted Regression Modeling for Predicting Trajectory Group Membership With Potentially-Modifiable Variables (Model 2)

A. Group 1: Minimal-user

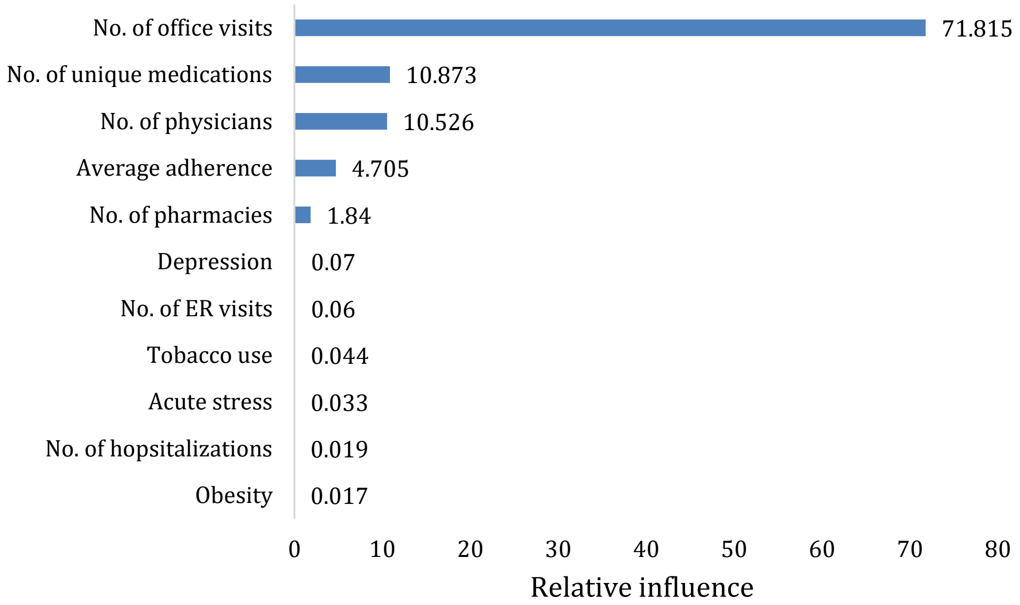

B. Group 2: Low-cost

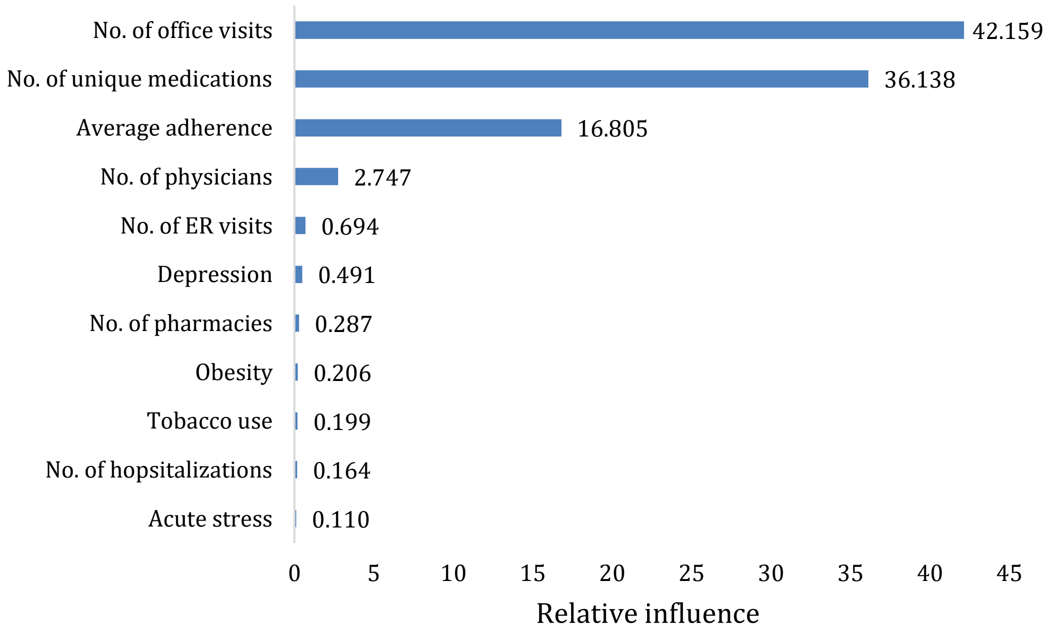

© 2020 Lauffenburger JC et al. *JAMA Network Open.*

C. Group 3: Rising-cost ("Cost-bloomer")

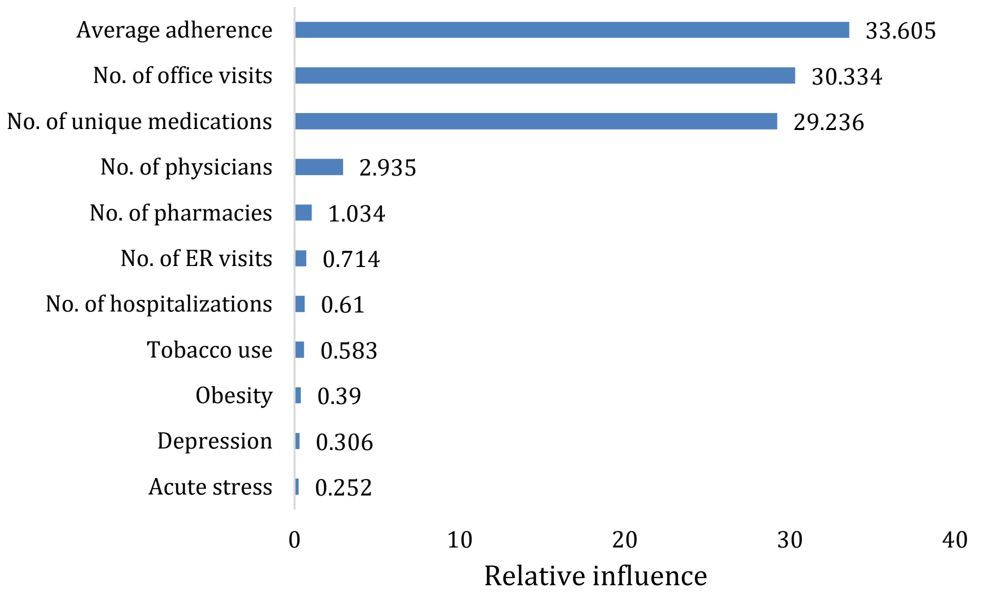

D. Group 4: Moderate-cost

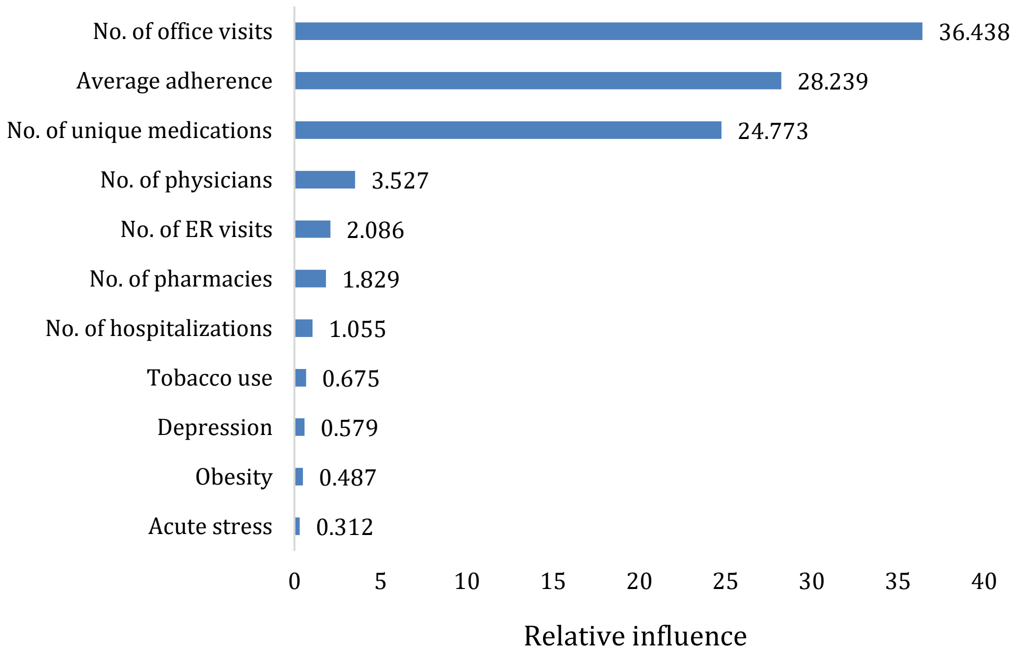

© 2020 Lauffenburger JC et al. *JAMA Network Open.*

E. Group 5: High-cost

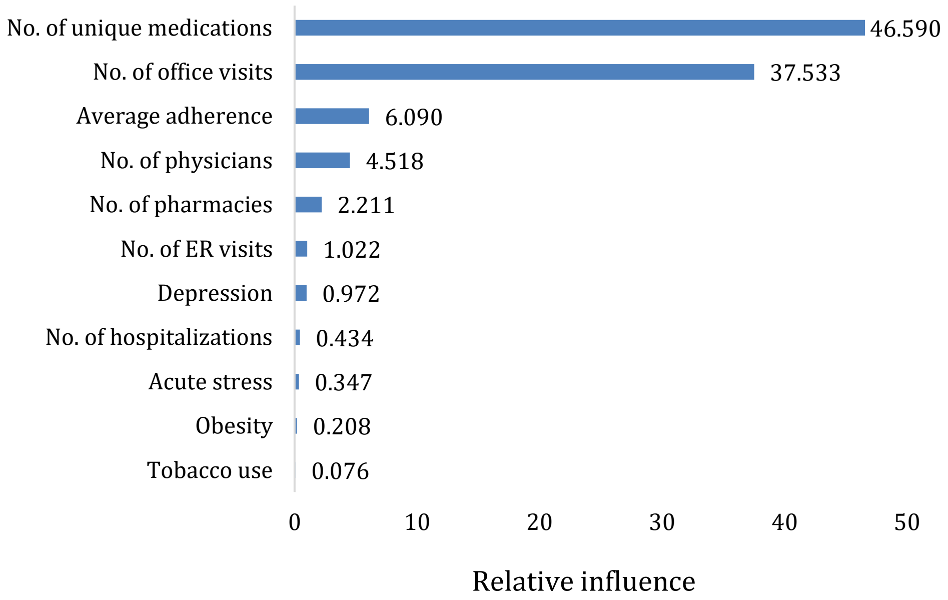

© 2020 Lauffenburger JC et al. *JAMA Network Open.*

eTable 4. Validation C-Statistics From Models Predicting Patients With Future Rising Spending

Model	Predictors	Cost-bloomers in Year 2* (n=20,470)	Group 3: Rising-cost (n=24,736)
3	All Year 0	0.667	0.764
4	Potentially-modifiable Year 0	0.643	0.753

*Defined by being in lower 90% in Year 1 and top 10% in Year 2

© 2020 Lauffenburger JC et al. *JAMA Network Open.*

eTable 5. Geographic Region and Baseline Chronic Condition Medication Classes By Trajectory Group

	Group 1: Minimal-user	Group 2: Low-cost	Group 3: Rising-cost	Group 4: Moderate-cost	Group 5: High-cost
**Geographic region of residence**			**N (%)**		
Midwest	8,512 (22.7)	11,890 (24.5)	6,313 (25.5)	20,612 (24.7)	31,123 (23.0)
Northeast	5,945 (15.8)	7,828 (16.1)	4,385 (17.7)	15,166 (18.2)	27,778 (20.5)
South	13,707 (36.5)	19,150 (39.4)	9,620 (38.9)	32,871 (39.4)	53,942 (39.9)
West	8,605 (22.9)	9,418 (19.4)	4,283 (17.3)	14,280 (17.1)	21,917 (16.2)
**Medication class adherence (PDC)**			**Mean (SD)**		
Alpha glucosidase inhibitors	0.14 (0.20)	0.69 (0.26)	0.53 (0.21)	0.73 (0.26)	0.71 (0.29)
ACEIs/ARBs	0.58 (0.31)	0.81 (0.24)	0.79 (0.27)	0.85 (0.22)	0.84 (0.23)
Anticholinergics, inhaled	0.52 (0.36)	0.47 (0.31)	0.58 (0.33)	0.60 (0.29)	0.67 (0.30)
Beta-blockers	0.59 (0.31)	0.80 (0.24)	0.79 (0.26)	0.85 (0.22)	0.85 (0.22)
Biguanide	0.55 (0.29)	0.72 (0.28)	0.70 (0.28)	0.81 (0.23)	0.82 (0.23)
Bisphosphonates	0.58 (0.28)	0.69 (0.28)	0.68 (0.28)	0.71 (0.28)	0.72 (0.28)
Calcium channel blockers	0.58 (0.32)	0.80 (0.26)	0.81 (0.25)	0.85 (0.22)	0.85 (0.23)
Dipeptidyl peptidase-4 inhibitors	0.55 (0.35)	0.66 (0.32)	0.68 (0.34)	0.73 (0.28)	0.79 (0.26)
Diuretics, thiazide	0.56 (0.31)	0.79 (0.26)	0.79 (0.26)	0.83 (0.23)	0.81 (0.25)
Leukotriene modulators	0.30 (0.25)	0.48 (0.31)	0.53 (0.29)	0.62 (0.33)	0.72 (0.30)
Long-acting beta-agonists	0.59 (0.30)	0.51 (0.39)	0.75 (0.32)	0.48 (0.28)	0.57 (0.33)
Meglitinides	-	0.79 (0.24)	0.57 (0.39)	0.71 (0.30)	0.70 (0.29)
Other anti-hypertensives	0.47 (0.30)	0.73 (0.31)	0.74 (0.30)	0.79 (0.27)	0.76 (0.30)
Selective estrogen receptor modulators	0.66 (0.26)	0.76 (0.28)	0.70 (0.29)	0.80 (0.24)	0.81 (0.24)
Statins	0.57 (0.30)	0.78 (0.25)	0.77 (0.26)	0.82 (0.22)	0.84 (0.22)
Sulfonylureas	0.59 (0.30)	0.73 (0.28)	0.70 (0.28)	0.83 (0.22)	0.83 (0.23)
Thiazolidinediones	0.48 (0.29)	0.63 (0.27)	0.59 (0.28)	0.69 (0.28)	0.73 (0.28)
Xanthines	0.45 (0.29)	0.73 (0.28)	0.69 (0.44)	0.71 (0.29)	0.74 (0.30)

Abbreviations: PDC, Proportion of Days Covered; ACEI/ARB, Angiotensin-converting enzyme inhibitors/angiotensin receptor blockers

© 2020 Lauffenburger JC et al. *JAMA Network Open.*

eTable 6. Validation C-Statistics From Sensitivity Analyses

Predictors	Group 1: Minimal-user	Group 2: Low-cost	Group 3: Rising-cost	Group 4: Moderate-cost	Group 5: High-cost
All baseline predictors (Model 1)+ Region	0.956	0.819	0.765	0.729	0.903
All baseline predictors (Model 1) with adherence by individual class	0.955	0.816	0.766	0.729	0.900
Potentially-modifiable predictors (Model 2) with adherence by individual class	0.946	0.785	0.756	0.689	0.874

© 2020 Lauffenburger JC et al. *JAMA Network Open.*

eFigure 5. Two-Year Spending Patterns Using Trajectory Modeling In 2013-2014 Data

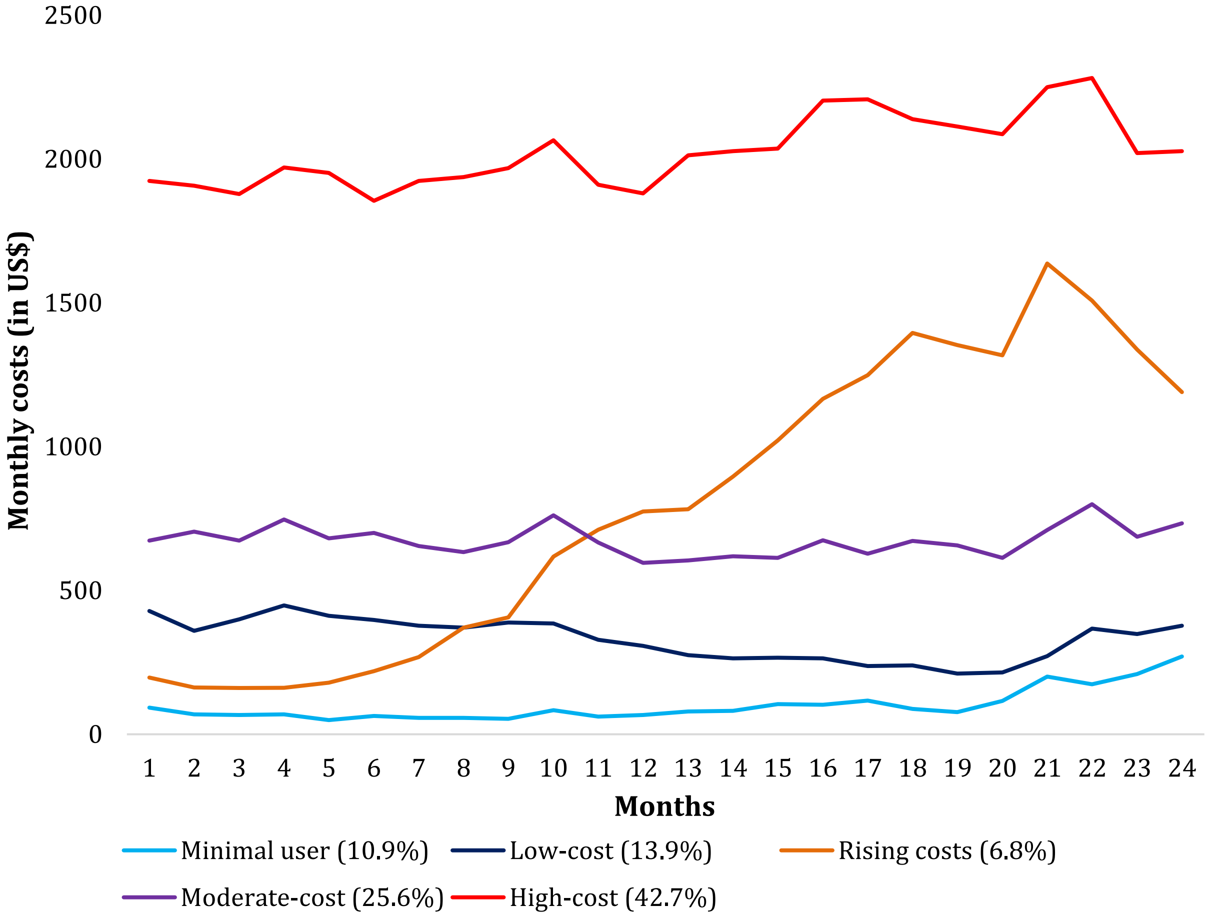

Note: The mean spending levels using 5-group trajectory modeling in the full 2013-2014 sample (n=297,150) are plotted. The percentages refer to the number of patients who belong to each trajectory group out of the full cohort.

© 2020 Lauffenburger JC et al. *JAMA Network Open.*

eTable 7. Ability of Models to Predict Two-Year Spending Trajectory Groups In 2013-2014 Data

**Group**	**Validation C-statistic**
**All baseline predictors**	**Model 1**
Group 1: Minimal-user	0.983
Group 2: Low-cost	0.864
Group 3: Rising-cost	0.812
Group 4: Moderate-cost	0.777
Group 5: High-cost	0.941
**Potentially-modifiable predictors**	**Model 2**
Group 1: Minimal-user	0.952
Group 2: Low-cost	0.787
Group 3: Rising-cost	0.767
Group 4: Moderate-cost	0.696
Group 5: High-cost	0.887

© 2020 Lauffenburger JC et al. *JAMA Network Open.*
